# Abdominal Drains Retrieved Laparoscopically 15 Years Post Laparotomy

**DOI:** 10.7759/cureus.1711

**Published:** 2017-09-24

**Authors:** Anant Dinesh, Vishnu R Mani, Aleksandr Kalabin, Irwin C White-Gittens, Brian Donaldson

**Affiliations:** 1 Department of General Surgery, Columbia University College of Physicians and Surgeons at Harlem Hospital Center; 2 Department of Surgery, New York University School of Medicine, and the Laura and Isaac Perlmutter Cancer Center, Columbia University School of Physicians and Surgeons at Harlem Hospital Center

**Keywords:** foreign body, drains, laparoscopy

## Abstract

A retained intra-abdominal foreign body is a common occurrence that is frequently underreported due to its medicolegal implications. Sponges, gauzes, surgical instruments, abdominal drains, etc. have been reported in the literature. The most common presentation for a retained intra-abdominal foreign body is postoperative abdominal infections or bleeding, frequently seen in the immediate postoperative period. Most of these foreign bodies are removed by exploratory laparotomy owing to recent abdominal surgeries or presentation as complicated abdominal masses. Here, we report a case with retained intra-abdominal drains for 15 years with minimal symptoms presenting as an intermittent abdominal pain; the drains were removed using laparoscopic intervention.

## Introduction

Retained intra-abdominal foreign bodies are not very uncommon phenomena but are generally under-reported due to their medicolegal implications. The most common foreign body seen after an abdominal surgery is the sponge, called gossypiboma. Here, we report retained intra-abdominal drains as the foreign body in the peritoneal cavity and the role of laparoscopic surgery in their retrieval. Informed consent was obtained from the patient for this study.

## Case presentation

**Figure 1:**
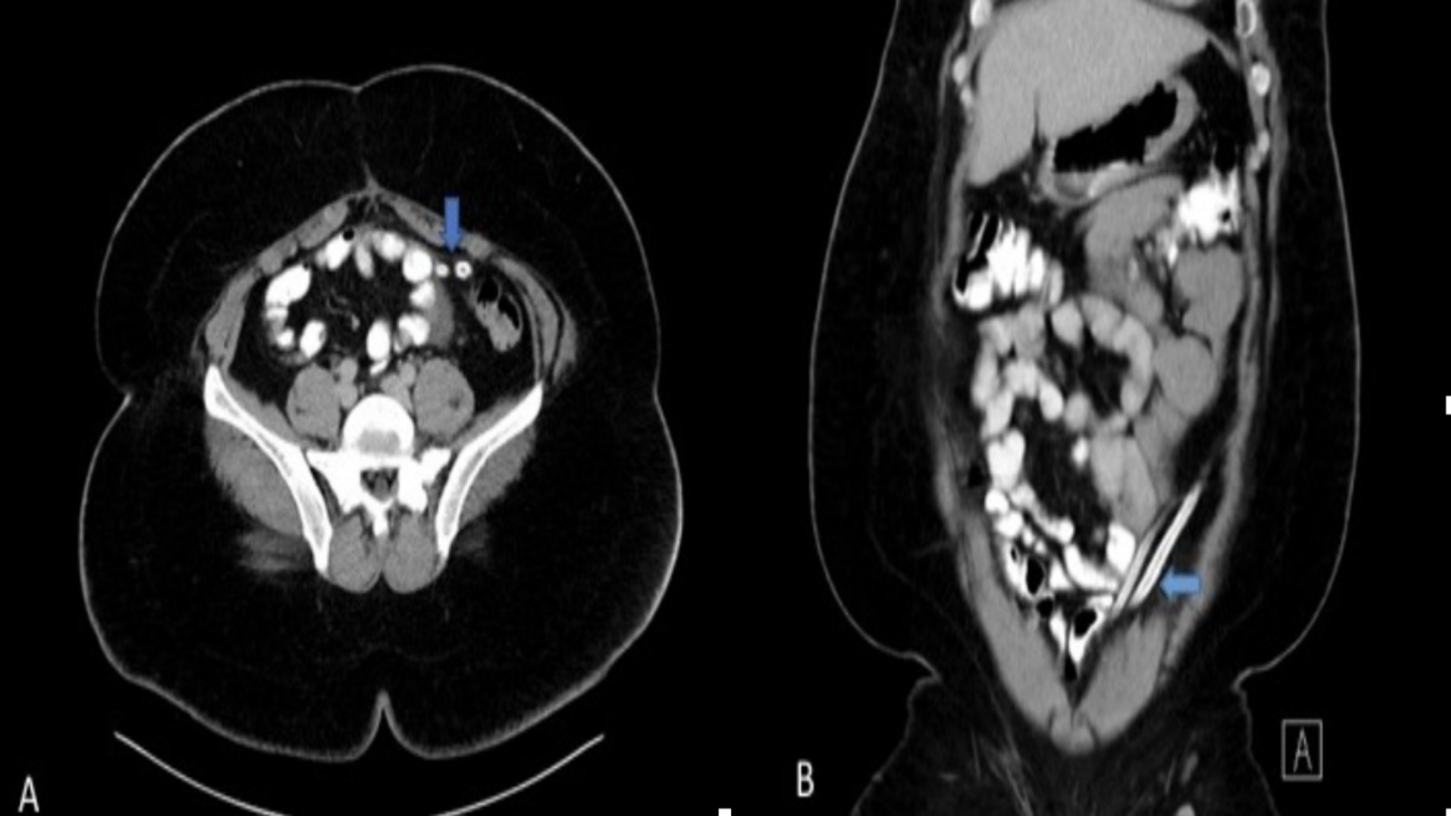
CT abdomen Computed tomography: CT

A 38-year-old male presented to the emergency room with a complaint of abdominal pain around the umbilicus for one day; it was associated with nausea and vomiting. He stated having similar episodes of pain intermittently for the last one year, and the pain was relieved with analgesics after a few hours. He didn’t report any fever, change in bowel habits, or blood in stool or vomitus. He had a past medical history of diabetes mellitus, which was well controlled with medications, and his past surgical history was significant for an exploratory laparotomy with bowel resection for a small bowel obstruction done in 2002 in his home country. His vitals were stable at presentation. On examination, his abdomen was soft with mild tenderness in the left side of the abdomen with no guarding or rigidity. Bowel sounds were heard and were normal. His laboratory workup was unremarkable. A computed tomography (CT) scan of the abdomen and pelvis suggested two blind-ending radio-dense drains/catheters coursing within the peritoneum with proximal tips in the left mid abdomen and distal tips located in the lower pelvis adjacent to the dome of the urinary bladder (Figure 1) The rest of the findings were unremarkable.

**Figure 2:**
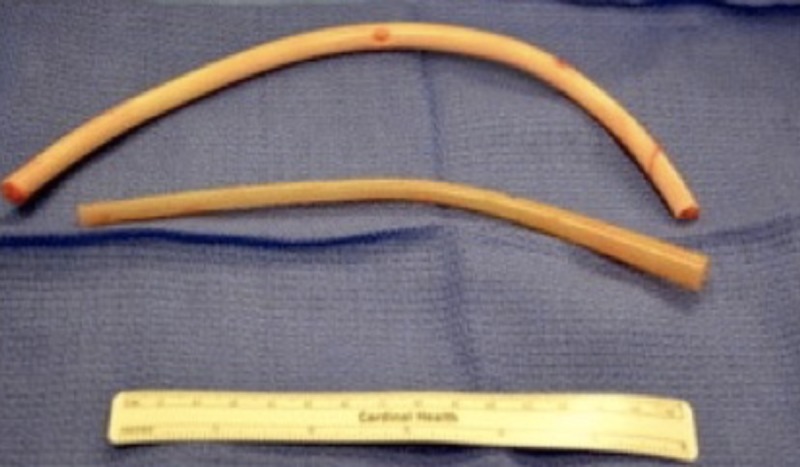
Retrieved drains

The patient was planned for the laparoscopic removal of the foreign body from the peritoneal cavity and informed consent was obtained. A diagnostic laparoscopy was performed using three 5 mm ports. The first port was placed at Palmer’s point using the Visiport technique, the other two 5 mm ports were placed under vision in the upper midline and the left anterior axillary line at the level of the umbilicus. Adhesions were found between the left abdominal wall and the omentum. Adhesiolysis was performed using LigaSure (Medtronics, Minnesota, United States) and a sharp dissection, and two pieces of drains were found entrapped between the adhesions and wrapped around the omentum. Careful dissection was performed and the drains were removed and retrieved through the 5 mm port (Figure 2). The peritoneal cavity was inspected for any injuries or bleeding. No bowel or visceral injuries were seen. The skin was closed after releasing the pneumoperitoneum and removing the ports. The procedure and postoperative course were uneventful. The patient was discharged from the hospital on postoperative day one.

## Discussion

Retained foreign bodies after abdominal surgeries have been reported in the literature and the most commonly reported are sponges or gauzes. The incidence of such occurrences is 1:5027, which may be an under-reported number [1]. The most common complications involved are intraperitoneal infections, perforation of the bowel, or injury to intra-abdominal viscera or vessels. Patients most commonly present with symptoms of abdominal pain, abdominal mass, bleeding, bowel obstruction, fever, diarrhea, and weight loss [2-4]. Generally, they present within a few weeks or months with these symptoms. Asymptomatic presentations have been reported in the literature; however, intraoperative findings suggested loculated infections [5]. In our case, the patient had a retained foreign body for 15 years with no obvious symptoms or signs of infections or injuries. Moreover, he had been asymptomatic for most of the duration since his initial surgery.

The role of laparoscopic surgery in the removal of such foreign bodies has been undermined owing to the recent presentation or detection of errors. Surgeons prefer to use the same incision for the removal of these foreign bodies after recent surgeries [6-7]. In delayed cases that most commonly present as an intra-abdominal mass or tumor, the open approach has been most commonly practiced owing to suspicion for malignancy and anticipated difficult surgical planes [5].

## Conclusions

The use of laparoscopy for such procedures is a helpful tool for avoiding inadvertent complications of exploratory laparotomy, and it allows early postoperative recovery and discharge. Hence, we urge the surgical community to consider laparoscopy as the procedure of choice, where appropriate, for the removal of foreign bodies from the abdominal cavity owing to the ease, minimal invasiveness, and decreased postoperative complications.
